# Muscle Strengthening Exercises for the Foot and Ankle: A Scoping Review Exploring Adherence to Best Practice for Optimizing Musculoskeletal Health

**DOI:** 10.1002/jfa2.70040

**Published:** 2025-04-03

**Authors:** John W. A. Osborne, Hylton B. Menz, Glen A. Whittaker, Matthew Cotchett, Karl B. Landorf

**Affiliations:** ^1^ Discipline of Podiatry School of Allied Health, Human Services and Sport La Trobe University Melbourne Australia

**Keywords:** ankle, foot, resistance training

## Abstract

**Background:**

Foot and ankle muscle strengthening exercises are common interventions for many musculoskeletal conditions that are associated with pain and limited function in the lower limb. The scientific literature has a multitude of strengthening exercises recommended, and they have been criticized for not adhering to best practice and for being poorly reported. The aims of this scoping review were to (i) describe what foot and ankle strengthening exercises have been recommended in the scientific literature, (ii) compare the prescription of these exercises to best practice recommendations, and (iii) assess the completeness of the reporting of these exercises and exercise programs.

**Methods:**

This scoping review was conducted in accordance with the Joanna Briggs Institute methodology for scoping reviews. A systematic search of peer‐review journal articles was conducted on 23 February 2023. Study designs that were included were experimental, quasi‐experimental, feasibility, pilot studies, and observational. For each study included in the review, study design and participant details such as age, sex, and conditions treated were noted. To describe the foot and ankle strengthening exercises, each exercise was noted, which included its name, the number of sets and repetitions recommended, the load type and its magnitude, and whether there were any progression strategies. Exercises were grouped according to primary movement and a general exercise descriptor. To compare to best practice, each program's prescription parameters of frequency, intensity, and time were compared to the *American College of Sports Medicine's* (ACSM) guidelines. To assess completeness of reporting, each study was assessed with the *Consensus on Exercise Reporting Template* (CERT).

**Results:**

The search yielded 1511 documents, and 87 were included after full‐text screening. Of the included studies, most were randomized controlled trials, and the most common participants were healthy adults (mean age range: 18–83 years). Across all studies, a total of 300 foot and ankle exercises were prescribed. The most common strengthening exercise category involved ankle plantar flexion (25% of 300 exercises), followed by plantar foot intrinsics (16%). The most common prescription of strengthening exercises included 3 sets (37%) of 10 repetitions (38%) performed 3 times per week (34%), often without a prescribed load (66%). Prescribed sets per muscle group met ACSM recommendations for novice lifters in 93% of studies. In contrast, load intensity (for increasing muscle strength) was prescribed at the recommended dose of 60% of 1 repetition maximum or greater in only 2% of exercises. The median score for completeness of reporting according to the CERT checklist was 31% of all items.

**Conclusions:**

This scoping review found that the studies predominantly included ankle plantar flexion and plantar foot intrinsic muscle strengthening exercises, typically prescribed at 3 sets of 10 repetitions, 3 times per week. When compared to best practice recommendations, load intensity in exercise prescription is commonly less than recommended or is not reported. In addition, the review highlights deficiencies in the reporting of exercise programs. We propose using established best‐practice exercise prescription guidelines like those from the ACSM and the adoption of CERT for reporting exercises in the scientific literature.

**Abbreviations**
ACSM: American College of Sports Medicine
CERT: Consensus on Exercise Reporting Template
Kg: Kilogram
RM: repetition maximum

## 1 Background

Foot and ankle muscle strengthening exercises are a common treatment modality for managing foot and ankle musculoskeletal conditions [1]. For example, strengthening exercises have been prescribed for several foot and ankle conditions to reduce pain and improve function [2, 3, 4]. They can also be used to improve strength for increased athletic performance and to provide general psychological benefits [5, 6, 7]. There are a multitude of strengthening exercises that are commonly prescribed, but there is ongoing debate about whether current exercise prescriptions effectively optimize strength gains [8, 9, 10, 11]. In addition, strengthening exercises, as well as exercises more generally, have been criticized for being poorly reported, which can lead to inconsistent prescription and poor reproducibility [12, 13, 14].

To ensure optimal exercise prescription, researchers and clinicians should consider how exercises are prescribed and the quality of their reporting. Fortunately, guidelines have been developed to provide structure for both the prescription and reporting of exercise [15, 16]. The *American College of Sports Medicine* (ACSM) has developed guidelines to provide recommendations for the optimization of strengthening exercise prescription [15]. These guidelines provide advice on the appropriate dosage of exercise training variables such as frequency (times per week), intensity (load type, magnitude, and progression), and time (sets and repetitions) to achieve maximal strength gains. In addition, for the reporting of exercises, the *Consensus on Exercise Reporting Template* (CERT)—a 16‐item minimum checklist to improve the reporting of exercise programs—was developed to improve transparency and reproducibility in clinical and research settings [16]. However, it is currently unclear whether exercises to strengthen the foot and ankle meet best practice guidelines or are being reported appropriately.

Therefore, the aims of this scoping review were to (i) describe what foot and ankle strengthening exercises have been recommended in the scientific literature, (ii) compare these exercise prescriptions to best practice recommendations, and (iii) assess the completeness of the reporting of these exercises and exercise programs.

## 2 Methods

### 2.1 Study Design

This study is a scoping review that utilized a systematic literature search. To provide a robust framework, the review was conducted in accordance with the Joanna Briggs Institute methodology for scoping reviews and is reported using the Preferred Reporting Items for Systematic Reviews and Meta‐Analyses Extension for Scoping Reviews [17, 18].

### 2.2 Eligibility Criteria

#### 2.2.1 Document Type

Studies needed to have been reported in a peer‐reviewed journal.

#### 2.2.2 Types of Studies

Study designs included the following: experimental and quasi‐experimental (including randomized controlled trials, nonrandomized controlled trials, and single‐group pre‐post intervention studies), feasibility and pilot studies (randomized and nonrandomized), and observational studies (including case series, case reports, case‐control, and cohort studies). Systematic reviews were not included; however, the original studies included in any systematic review were considered for inclusion.

#### 2.2.3 Participants

Studies were included if foot and ankle exercises were prescribed as part of a strengthening program for adults (aged 18 years or over) with or without musculoskeletal conditions or injury, and the program was prescribed for 1 week or longer. Studies were excluded if participants had chronic or systemic neurological or cardiovascular disorders, such as diabetes, neuropathy, and Charcot–Marie–Tooth disease.

#### 2.2.4 Types of Strengthening Exercises

Studies that provided foot and ankle strengthening exercises across multiple sessions were included. Studies where an exercise was prescribed for immediate pre‐post testing (e.g., assessing the intrinsic muscle activation during or immediately after the performance of the short foot exercise) or used special equipment that is not readily utilized in clinical practice (e.g., isokinetic strengthening machines) were excluded. If a study mentioned that rehabilitation was performed without specifying “strengthening” or the exercises themselves, these were also excluded. All foot and ankle strengthening exercises in a study were included in the analysis.

### 2.3 Search Strategy and Information Sources

**FIGURE 1:**
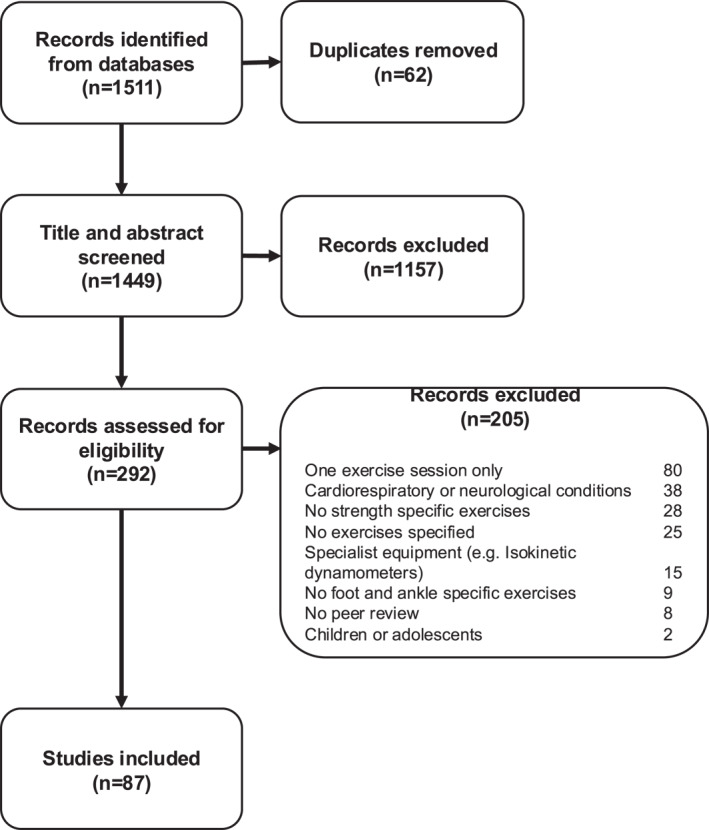
PRISMA (Preferred Reporting Items for Systematic Reviews and Meta‐Analyses) flowchart illustrating the step‐by‐step process leading to identification of studies eligible for the review.

A systematic search was conducted on 23 February 2023 (Supporting Information S1). The Ovid platform was used to search Ovid MEDLINE (1946‐present) and CINAHL (1980‐present). To broaden the search, some terms were truncated with wildcard symbols. All keywords were searched in title, book title, abstract, original title, name of substance word, subject heading word, floating subheading word, keyword heading word, organism supplementary concept word, protocol supplementary concept word, rare disease supplementary concept word, unique identifier, and synonyms. All titles and abstracts found in the search were imported to Zotero 6.0 (Digital Scholar, Vienna, Virginia, USA) for inclusion assessment. After duplicate removal, articles were then assessed based on the title and abstract by the primary author (J.W.A.O.) for inclusion in the review. Articles deemed appropriate for inclusion had the full text obtained and reviewed for eligibility requirements. Once eligibility was determined, each article was analyzed and relevant data extracted by the primary author with spot checks by two other reviewers (M.P.C. and K.B.L.). A PRISMA flow diagram (Figure 1) is included to document the phases of the systematic review. Gray literature and the reference lists of systematic reviews and articles that met inclusion criteria were also hand‐searched for additional relevant articles.

### 2.4 Samples and Exercise Descriptions (including Definitions)

Data were extracted from studies included in the review by the primary author (JWAO) using Microsoft Excel (Microsoft Corporation, Redmond, Washington, USA). The data included details about the study design, population targeted for recruitment, conditions treated, mean age, and sex of participants. Exercise data were extracted defining the exercises prescribed, including the name of the exercise. The primary author then grouped exercises by context to enable categorization of the primary movement and a general exercise descriptor. Further exercise prescription details were extracted including frequency (exercise frequency and duration of program), intensity (load intensity, load type, and progression of exercise), and time (sets, repetitions, etc.).

#### 2.4.1 Populations Targeted

The population targeted for recruitment into each study was documented as stated in each article's text.

#### 2.4.2 Conditions Treated

The conditions treated needed to be specified in the text. Specific conditions were grouped according to broad categories. For example, midportion and insertional Achilles tendinopathy were grouped as “Achilles tendinopathy” and chronic and functional ankle instability were grouped as “ankle instability”.

#### 2.4.3 Primary Movement

Exercises were grouped according to the primary movement of the joint during the exercise. This included exercises for the ankle (plantar flexion, dorsiflexion, inversion, or eversion) or the digits (flexion, extension, adduction, and abduction). Exercises where more than one movement was needed were considered “multiple” (i.e., multiple movements). Exercises without movement occurring at a joint (e.g., standing still) were considered “static”.

#### 2.4.4 Muscle Group

To calculate sets per muscle group, we determined that all exercises performing a primary movement, (e.g., ankle plantar flexion or ankle inversion), would be considered one muscle group. Therefore, if a study had three ankle plantar flexion exercises where the participant performed three sets of each, it was considered nine sets for the ankle plantar flexion muscle group.

#### 2.4.5 Exercise Categories

We grouped exercises by category to provide them with context relative to their intended aim. These categories included targeted movements (ankle plantar flexion, ankle dorsiflexion, ankle inversion, and ankle eversion), specific structures of interest (plantar intrinsics, hallux, and lesser digits), and the desired outcome (balance and dexterity) of the exercise. For example, resisted ankle inversion targets the movement of ankle inversion, the short foot exercise targets the specific structures of the plantar intrinsics, and scrunching digits and writing the alphabet with the foot target the outcome of improving the dexterity of the digits, foot, and ankle.

#### 2.4.6 Frequency

A tally of the program's training frequency (e.g., to perform the program or exercise three times a week) was documented. In some studies, different exercises in a program were performed at different frequencies, so the frequency was documented per exercise rather than per study.

#### 2.4.7 Duration of Program

The duration of the program (e.g., an 8‐week exercise program) was documented.

#### 2.4.8 Intensity (Load Intensity)

The intensity was also quantified as a percentage of repetition maximum (RM), and/or the raw value provided was documented. When a weight or resistance band type was provided with no context of its RM, it was only documented as the weight specified.

#### 2.4.9 Load Type

Load types were documented, which included any equipment that created a resistance or load for the exercise (e.g., Thera‐Band, bodyweight, and weighted bags). Where nothing was specified, “not reported” was documented.

#### 2.4.10 Progression of Exercise

Exercise progressions were documented and categorized as “increasing in volume only,” “increasing in load only,” “increasing in volume then load,” “increasing in load then volume,” “increasing in load and volume,” an “alternative” method that was neither load or volume, an “unspecified” method of progressing exercise, or “not reported.”

#### 2.4.11 Time (Sets and Repetitions)

The tally of sets and repetitions prescribed for each exercise was documented independently. The repetition range (e.g., heel raises for 10 repetitions), time (e.g., inverting the ankle for 10 min), and distance (e.g., scrunching a 1‐m length of towel) were also documented.

### 2.5 Comparison of Exercise Prescription to the ACSM Guidelines Recommendations

Comparison was made to the ACSM guidelines for exercise testing and prescription, which provide recommendations for the best dosage of strengthening exercise variables (e.g., frequency, intensity, and time) to achieve strength gains in the following groups: novice, intermediate, and advanced lifters [15]. The term “lifter(s)” refers to anyone performing resistance strength training; a novice lifter is someone who has little to no experience with strength training and an advanced lifter is someone who performs strength training regularly and has years of experience [15]. The guidelines were summarized to compare each study against best practice (Table 1). The summary provides the optimal number of sets per muscle group, load, or resistance relative to repetition maximum and the frequency of performing the exercise. Groups with different amounts of training experience have different recommendations for achieving optimal strength gains [15].

**TABLE 1:** Best practice exercise variable dosage recommendations for muscle strength as recommended by the ACSM guidelines [15].

Group denomination	Exercise prescription variables		
Experience level	Frequency	Intensity (load intensity per exercise)	Time (sets)
Novice	2–3 days per week	60%–70% 1RM^a^ (8–12 RM^a^)	1–3 per muscle group
Intermediate and advanced lifters	2–3 days per week	80%–100% 1RM^a^ (1–6 RM^a^)	Multiple sets to be used with systematic variation of volume and intensity over time.

*Note:* This table outlines the recommended sets, load intensity and frequency of performing exercises for strength gains for the three levels of experience.

^a^ RM, repetition maximum.

When comparing exercise prescription variables to the ACSM guidelines, the following structures were used. For repetitions, the prescribed dosage for each exercise was compared to the ACSM guidelines. For sets per muscle group, all exercises within each study were grouped according to primary movements (e.g., all ankle plantar flexion exercises were grouped together) and then the collective sets were compared against the ACSM guidelines. For load intensity and training frequency, the prescribed dose of each individual exercise was compared against the ACSM guidelines. Finally, every program within each study was compared to best practice recommendations to ascertain if it met all three domains of sets, recommended load intensity, and frequency.

### 2.6 Quality of Reporting

The primary author (JWAO) assessed the quality of reporting of exercises in each study using the CERT checklist [16]. The CERT is a 16‐item checklist developed by an international panel of experts, and was designed to improve the transparency and reproducibility of exercise programs used in scientific studies [16].

## 3 Results

### 3.1 Characteristics of Included Studies

The search yielded 1511 documents; after removing 62 duplicates, 1449 documents were screened by the title and abstract. Following the title and abstract review, there were 292 relevant documents, and 87 documents were included after a full‐text screening (Table 2). Most studies were randomized controlled trials [2, 4, 8, 19, 22, 23, 24, 27, 29, 31, 32, 34, 35, 36, 37, 39, 40, 43, 44, 45, 46, 47, 51, 52, 53, 55, 56, 57, 58, 59, 60, 62, 63, 65, 66, 67, 69, 71, 72, 73, 74, 75, 77, 82], followed by pre‐post intervention studies [20, 26, 28, 38, 42, 49, 61, 64, 68, 70, 76, 84, 87, 90, 91, 95, 96]. Also included were trial protocols [30, 33, 54, 79, 94], feasibility studies [80, 93, 101], nonrandomized controlled trials [21, 41, 48, 78], pilot studies [50, 83], case reports [25, 86], and a case series [81]. Where both a protocol and a trial were available, either the protocol or trial was used for results depending on which provided more details.

**TABLE 2:** Characteristics of included studies.

Study details				Study participant details			
Authors	Title	Year	Design	Population	Condition treated	Mean age, yrs	% Female
Abdalbary [19]	Foot mobilization and exercise program combined with toe separator improve outcomes in women with moderate hallux valgus at a 1‐year follow‐up randomized clinical trial	2018	Randomized controlled trial	Adults: Female	Hallux valgus	46	100%
Amaha et al. [20]	Effect of toe exercises and toe grip strength on the treatment of primary metatarsalgia	2020	Pre‐post intervention study	Adults (> 20 years)	Metatarsalgia	63	82%
Bae and Cho [21]^a^	Effects of community‐based comprehensive fall prevention program on muscle strength, postural balance, and fall efficacy in elderly people	2014	Non‐randomized controlled trial	Adults: Older (65+ yrs)	Healthy	74	97%
Bagherian et al. [22]	Corrective exercises improve movement efficiency and sensorimotor function but not fatigue sensitivity in chronic ankle instability patients: A randomized controlled trial	2019	Randomized controlled trial	Athletes: College	Ankle instability	21	0%
Bassett and Prapavessis [23]	Home‐based physical therapy intervention with adherence‐enhancing strategies versus clinic‐based management for patients with ankle sprains	2007	Randomized controlled trial	Adults	Ankle injuries: Acute and chronic	30	40%
Bleakley et al. [24]	Effect of accelerated rehabilitation on function after ankle sprain: A randomized controlled trial	2010	Randomized controlled trial	Adults	Ankle injuries: Acute and chronic	26	31%
Çil et al. [25]	Outpatient versus home management protocol results for plantar fasciitis	2019	Case report	Adults	Plantar heel pain	49	74%
Docherty et al. [26]	Effects of strength training development and joint position sense in functionally unstable ankles	1998	Pre‐post intervention study	College/University students	Ankle instability	21	50%
Dogra and Rangan [27]	Early mobilization versus immobilization of surgically treated ankle fractures. Prospective randomized control trial	1999	Randomized controlled trial	Adults	Ankle fracture	43	52%
Feltner et al. [28]	Strength training effects on rearfoot motion in running	1994	Pre‐post intervention study	Athletes: runners	Healthy	19	61%
Franettovich Smith et al. [29]	A comparison of a rigid tape and exercise, elastic tape and exercise and exercise alone on pain and lower limb function in individuals with exercise‐related leg pain: A randomized controlled trial	2014	Randomized controlled trial protocol	Adults: Female	Lower limb pain	n/a^b^	100%
Franettovich Smith et al. [30]	Foot exercise plus education versus wait and see for the treatment of plantar heel pain (FEET trial): A protocol for a feasibility study	2020	Feasibility study protocol	Adults	Plantar heel pain	n/a^b^	n/a
Gatz et al. [31]	Eccentric and isometric exercises in Achilles tendinopathy evaluated by the VISA‐A score and shear wave elastography	2020	Randomized controlled trial	Adults	Achilles tendinopathy	50	36%
Gras et al. [32]	A comparison of hip versus ankle exercises in elders and the influence on balance and gait	2004	Randomized controlled trial	Adults	Healthy	74	19%
Habets et al. [33]	Alfredson versus Silbernagel exercise therapy in chronic midportion Achilles tendinopathy: Study protocol for a randomized controlled trial	2017	Randomized controlled trial protocol	Athletes: Recreational	Achilles tendinopathy	n/a^b^	n/a^b^
Hale et al. [34]	The effect of a 4‐week comprehensive rehabilitation program on postural control and lower extremity function in individuals with chronic ankle instability	2007	Randomized controlled trial	Adults	Ankle instability	21	58%
Hall et al. [35]	Balance‐ and strength‐training protocols to improve chronic ankle instability deficits: A randomized controlled trial	2015	Randomized controlled trial	Adults	Ankle instability	19	56%
Han et al. [36]	Effects of a 4‐week exercise program on balance using elastic tubing as a perturbation force for individuals with a history of ankle sprains	2009	Randomized controlled trial	Adults	Ankle instability	21	50%
Hartmann et al. [37]	The effect of a foot gymnastic exercise program on gait performance in older adults: A randomized controlled trial	2003	Randomized controlled trial	Adults: Older (66+ yrs)	Healthy	76	64%
Hashimoto and Sakuraba [38]	Strength training for the intrinsic flexor muscles of the foot: Effects on muscle strength, the foot arch, and dynamic parameters before and after the training	2014	Pre‐post intervention study	Adults	Healthy	29	0%
Heide et al. [39]	The effectiveness of radial extracorporeal shock wave therapy (rESWT), sham‐rESWT, standardized exercise program or usual care for patients with plantar fasciopathy: Study protocol for a double‐blind, randomized, and sham‐controlled trial	2020	Randomized controlled trial protocol	Adults	Plantar heel pain	n/a^b^	n/a^b^
Houck et al. [40]	Randomized controlled trial comparing orthosis augmented by either stretching or stretching and strengthening for stage II tibialis posterior tendon dysfunction	2015	Randomized controlled trial	Adults	Tibialis posterior tendinopathy	58	78%
Hultman et al. [41]	The effect of early physiotherapy after an acute ankle sprain	2010	Non‐randomized controlled trial	Adults	Ankle injuries: Acute and chronic	35	46%
Jonsson et al. [42]	New regimen for eccentric calf‐muscle training in patients with chronic insertional Achilles tendinopathy: Results of a pilot study	2008	Pre‐post intervention study	Adults	Achilles tendinopathy	53	56%
Jung et al. [43]	Effect of foot orthoses and short‐foot exercise on the cross‐sectional area of the abductor hallucis muscle in subjects with pes planus: A randomized controlled trial	2011	Randomized controlled trial	Individuals with pes planus	Healthy	22	n/r^c^
Kalaycioglu et al. [44]	The effectiveness of different ankle strengthening training programs on performance	2022	Randomized controlled trial	Adults: Male sedentary	Healthy	21	0%
Kaminski [45]	Effect of strength and proprioception training on eversion to inversion strength ratios in subjects with unilateral functional ankle instability	2003	Randomized controlled trial	Adults	Ankle instability	22	42%
Kamonseki et al. [46]	Effect of stretching with and without muscle strengthening exercises for the foot and hip in patients with plantar fasciitis: A randomized controlled single‐blind clinical trial	2016	Randomized controlled trial	Adults	Plantar heel pain	46	79%
Kim and Heo [47]	Comparison of virtual reality exercise versus conventional exercise on balance in patients with functional ankle instability: A randomized controlled trial	2019	Randomized controlled trial	College/University students	Ankle instability	21	76%
Kim and Lee [48]	The effect of short‐foot exercise using visual feedback on the balance and accuracy of knee joint movement in subjects with a flexible flatfoot	2020	Non‐randomized controlled trial	Individuals with pes planus	Healthy	22	47%
Kim et al. [49]	Effects of a 4‐week short‐foot exercise program on gait characteristics in patients with stage II posterior tibial tendon dysfunction	2021	Pre‐post intervention study	Adults	Tibialis posterior tendinopathy	22	47%
Kim et al. [50]	Aquatic versus land‐based exercises as early functional rehabilitation for elite athletes with acute lower extremity ligament injury: A pilot study	2010	Randomized pilot study	Athletes	Ankle injuries: Acute and chronic	26	27%
Kısacık et al. [51]	Short foot exercises have additional effects on knee pain, foot biomechanics, and lower extremity muscle strength in patients with patellofemoral pain	2021	Randomized controlled trial	Individuals with pes planus	Patellofemoral joint pain	42	n/r^c^
Kosik et al. [52]	Comparison of two rehabilitation protocols on patient‐ and disease‐oriented outcomes in individuals with chronic ankle instability	2017	Randomized controlled trial	Adults	Ankle instability	22	39%
Külünkoğlu et al. [53]	A comparison of the effectiveness of splinting, exercise, and electrotherapy in women patients with hallux valgus: A randomized clinical trial	2021	Randomized controlled trial	Adults: Female	Hallux valgus	48	100%
Lai et al. [54]	Effects of intrinsic‐foot‐muscle exercise combined with the lower extremity resistance training on postural stability in older adults with fall risk: Study protocol for a randomized controlled trial	2021	Randomized controlled trial protocol	Adults: Older (60+ yrs)	Healthy	n/a^b^	n/a^b^
Lee and Choi [55]	Effects of a 6‐week intrinsic foot muscle exercise program on the functions of intrinsic foot muscle and dynamic balance in patients with chronic ankle instability	2019	Randomized controlled trial	Adults	Ankle instability	21	67%
Lee et al. [56]	Short‐foot exercise promotes quantitative somatosensory function in ankle instability: A randomized controlled trial	2019	Randomized controlled trial	Adults	Ankle instability	22	50%
Lehtonen et al. [57]	Use of a cast compared with a functional ankle brace after operative treatment of an ankle fracture	2003	Randomized controlled trial	Post‐surgical patients	Ankle fracture	41	38%
Liu‐Ambrose et al. [58]	Resistance and agility training reduce fall risk in women aged 75–85 with low bone mass: A 6‐month randomized, controlled trial	2004	Randomized controlled trial	Adults: Older (75+ yrs)	Healthy	79	100%
Lynn et al. [59]	Differences in static‐ and dynamic‐balance task performance after 4 weeks of intrinsic‐foot‐muscle training: The short‐foot exercise versus the towel‐curl exercise	2012	Randomized controlled trial	Adults	Healthy	23	50%
Ma et al. [60]	Effects of combining high‐definition transcranial direct current stimulation with short‐foot exercise on chronic ankle instability: A pilot randomized and double‐blinded study	2020	Randomized controlled trial	Adults: Young	Ankle instability	21	54%
Mahmoud [61]	Examining the efficacy of short foot exercises as an effective stand‐alone treatment for mechanical low back pain associated with foot overpronation	2022	Pre‐post intervention study	Adults: Male	Low back pain	49	0%
Mansur et al. [62]	Shockwave therapy plus eccentric exercises versus isolated eccentric exercises for Achilles insertional tendinopathy: A double‐blinded randomized clinical trial	2021	Randomized controlled trial	Adults	Achilles tendinopathy	53	49%
Masood et al. [63]	Effects of 12‐week eccentric calf muscle training on muscle‐tendon glucose uptake and SEMG in patients with chronic Achilles tendon pain	2014	Randomized controlled trial	Adults	Achilles tendinopathy	28	30%
Matsumoto et al. [64]	Intrinsic foot muscle training affects plantar pressure distribution during a single‐group clinical trial	2019	Pre‐post intervention study	Adults	Healthy	20	50%
Mazloum and Sahebozamanir [65]	The effects Kinesiotaping and proprioceptive exercises in the rehabilitation management of volleyball players with chronic ankle instability	2016	Randomized controlled trial	Athletes: Volleyball	Ankle instability	23	n/r^c^
Mickle et al. [4] ^d^	Efficacy of a progressive resistance exercise program to increase toe flexor strength in older people	2016	Randomized controlled trial	Adults: Older (60+ yrs)	Healthy	69	80%
Mølgaard et al. [66]	Foot exercises and foot orthoses are more effective than knee focused exercises in individuals with patellofemoral pain	2018	Randomized controlled trial	Adults	Patellofemoral joint pain	31	70%
Moon and Jung [67]	Effect of incorporating short‐foot exercises in the balance rehabilitation of flat foot: A randomized controlled trial	2021	Randomized controlled trial	Individuals with pes planus	Healthy	21	56%
Mulligan and Cook [68]	Effect of plantar intrinsic muscle training on medial longitudinal arch morphology and dynamic function	2013	Pre‐post intervention study	Adults	Healthy	26	86%
Nilsson et al. [69]	Effects of a training program after a surgically treated ankle fracture: A prospective randomized controlled trial	2009	Randomized controlled trial	Post‐surgical patients	Ankle fracture	42	59%
Nunes et al. [70]	Different foot positionings during calf training to induce portion‐specific gastrocnemius muscle hypertrophy	2020	Pre‐post intervention study	Adults	Healthy	23	0%
Okamura et al. [71]	Effects of plantar intrinsic foot muscle strengthening exercise on static and dynamic foot kinematics: A pilot randomized controlled single‐blind trial in individuals with pes planus	2020	Randomized controlled trial	Individuals with pes planus	Healthy	20	85%
Öztürk and Çeli̇k [72]	Activity‐oriented exercise intervention for hallux valgus deformity in women	2022	Randomized controlled trial	Adults: Female	Hallux valgus	38	100%
Pabón‐Carrasco et al. [73]	Randomized clinical trial: The effect of exercise of the intrinsic muscle on foot pronation	2020	Randomized controlled trial	Adults	Healthy	20	53%
Petersen et al. [74]	Chronic Achilles tendinopathy: A prospective randomized study comparing the therapeutic effect of eccentric training, the AirHeel brace, and a combination of both	2007	Randomized controlled trial	Adults	Achilles tendinopathy	43	40%
Plaza‐Manzano et al. [75]	Manual therapy in joint and nerve structures combined with exercises in the treatment of recurrent ankle sprains: A randomized, controlled trial	2016	Randomized controlled trial	Athletes: Recreational	Ankle instability	24	30%
Powers et al. [76]	Six weeks of strength and proprioception training does not affect muscle fatigue and static balance in functional ankle instability	2004	Pre‐post intervention study	Adults	Ankle instability	22	42%
Ramachandra et al. [77]	Effect of intrinsic and extrinsic foot muscle strengthening exercises on foot parameters and foot dysfunctions in pregnant women: A randomized controlled trial	2018	Randomized controlled trial	Adults: Female	Healthy	28	100%
Rathleff et al. [2]	High‐load strength training improves outcome in patients with plantar fasciitis: A randomized controlled trial with 12‐month follow‐up	2014	Randomized controlled trial	Adults	Plantar heel pain	46	66%
Ribeiro et al. [78]	Impact of low cost strength training of dorsi‐ and plantar flexors on balance and functional mobility in institutionalized elderly people	2009	Randomized controlled trial	Adults: Older (70+ yrs)	Healthy	79	67%
Riel et al. [79]	Corticosteroid injection plus exercise versus exercise, beyond advice and a heel cup for patients with plantar fasciopathy: Protocol for a randomized clinical superiority trial (the FIX‐heel trial)	2020	Randomized controlled trial protocol	Adults	Plantar heel pain	n/a^b^	n/a^b^
Riel et al. [80]	Heavy‐slow resistance training in addition to an ultrasound‐guided corticosteroid injection for individuals with plantar fasciopathy: A feasibility study	2019	Randomized feasibility trial	Adults	Plantar heel pai	52	80%
Robinson et al. [81]	Nonsurgical approach in management of tibialis posterior tendinopathy with combined radial shockwave and foot core exercises: A case series	2020	Case series	Adults	Tibialis posterior tendinopathy	34	70%
Roller et al. [82]	Pilates reformer exercises for fall risk reduction in older adults: A randomized controlled trial	2017	Randomized controlled trial	Adults: Older (65+ yrs)	Balance, falls and mobility impairment	78	69%
Rowlands and Plumb [83]	The effects of a 4‐week barefoot exercise intervention on plantar pressure, impact, balance, and pain in injured recreational runners: a pilot study	2019	Randomized pilot study	Athletes: Runners	Healthy	31	38%
Schoenfelder [84]	A fall prevention program for elderly individuals: Exercise in long‐term care settings	2000	Pre‐post intervention study	Adults: Older (65+ yrs)	Healthy	83	75%
Schoenfelder and Rubenstein [85]	An exercise program to improve fall‐related outcomes in elderly nursing home residents	2004	Randomized controlled trial	Adults: Older (64+ yrs)	Healthy	75	100%
Senécal and Richer [86]	Conservative management of posterior ankle impingement: A case report	2016	Case report	Athletes: Recreational	Posterior impingement	37	0%
Simoneau et al. [87]	Adaptations to long‐term strength training of ankle joint muscles in old age	2007	Pre‐post intervention study	Adults: Older (70+ yrs)	Healthy	77	52%
Smith et al. [88]	Ankle strength and force sense after a progressive, 6‐week strength‐training program in people with functional ankle instability	2012	Randomized controlled trial	College/University students	Ankle instability	21	50%
Spink et al. [89]	Effectiveness of a multifaceted podiatry intervention to prevent falls in community dwelling older people with disabling foot pain: Randomized controlled trial	2011	Randomized controlled trial	Adults: Older (65+ yrs)	Foot pain	74	50%
Suciu et al. [90]	Gait analysis and functional outcomes after 12‐week rehabilitation in patients with surgically treated ankle fractures	2016	Pre‐post intervention study	Post‐surgical patients	Ankle fracture	50	47%
Sulowska‐Daszyk et al. [91]	Impact of short‐foot muscle exercises on quality of movement and flexibility in amateur runners	2020	Pre‐post intervention study	Athletes: Runners	Healthy	32	29%
Taddei et al. [92]	Effects of a foot strengthening program on foot muscle morphology and running mechanics: A proof‐of‐concept, single‐blind randomized controlled trial	2020	Randomized controlled trial	Athletes: Runners	Healthy	42	50%
Taddei et al. [93]	Effects of a therapeutic foot exercise program on injury incidence, foot functionality, and biomechanics in long‐distance runners: Feasibility study for a randomized controlled trial	2018	Feasibility study	Athletes: Runners	Healthy	42	42%
Treacy et al. [94]	Balance circuit classes to improve balance among rehabilitation inpatients: A protocol for a randomized controlled trial	2013	Randomized controlled trial protocol	Individuals who have had surgery	Balance, falls and mobility impairment	n/a^b^	n/a^b^
Tsuyuguchi et al. [95]	The effects of toe grip training on physical performance and cognitive function of nursing home residents	2019	Pre‐post intervention study	Adults: Older (70+ yrs)	Healthy	82	52%
Unver et al. [96]	Effects of short‐foot exercises on foot posture, pain, disability, and plantar pressure in pes planus	2020	Pre‐post intervention study	Individuals with pes planus	Healthy	21	63%
Van Reijen et al. [97]	The “strengthen your ankle” program to prevent recurrent injuries: A randomized controlled trial aimed at long‐term effectiveness	2017	Randomized controlled trial	Athletes	Ankle instability	38	50%
Vioreanu et al. [98]	Early mobilization in a removable cast compared with immobilization in a cast after operative treatment of ankle fractures: A prospective randomized study	2007	Randomized controlled trial	Post‐surgery	Ankle fracture	36	31%
Wright et al. [99]	A randomized controlled trial comparing rehabilitation efficacy in chronic ankle instability	2017	Randomized controlled trial	College/University students	Ankle instability	22	72%
Wright and Linens [100]	Patient‐reported efficacy 6 months after a 4‐week rehabilitation intervention in individuals with chronic ankle instability	2017	Randomized controlled trial	Adults	Ankle instability	20	86%
Yildiz et al. [101]	Intensive physiotherapy versus home‐based exercise and custom‐made orthotic insoles in patients with plantar fasciitis: Pilot study	2022	Randomized feasibility trial	Adults	Plantar heel pain	40	67%
Yıldırım Șahan et al. [102]	Comparison of short‐term effects of virtual reality and short foot exercises in pes planus	2021	Randomized controlled trial	Individuals with pes planus	Healthy	23	58%
Zhang et al. [103]	Acupuncture for chronic Achilles tendinopathy: A randomized controlled study	2013	Randomized controlled trial	Adults	Achilles tendinopathy	51	60%

^a^ The article was predominantly written in Korean, but the abstract and tables were in English, which provided sufficient information to extract the data needed for this scoping review.

^b^ Not applicable as it was part of a study protocol.

^c^ Not reported in the study.

^d^ This trial had two intervention groups that were randomized and a third (control) group that was not randomized.

#### 3.1.1 Populations Targeted

The mean age across studies was 39 years with a range of 18–83 years. Of the 87 studies, 38 (44%) used a general population of adults [2, 10, 20, 23, 24, 25, 27, 30, 31, 32, 34, 35, 36, 38, 39, 40, 41, 42, 45, 46, 49, 52, 55, 56, 59, 62, 63, 64, 66, 70, 73, 76, 79, 80, 81, 100, 101, 103], where specific subgroups of adults were used in 49 studies, including older adults in 12 of 87 studies (14%) [4, 21, 37, 54, 58, 78, 82, 84, 85, 87, 89, 95], females only in 5 (6%) [19, 29, 53, 72, 77] and males only [61], male sedentary adults [44], and young adults (between the ages of 18–30 years) [60] in 1 (1%) each. Twelve of 87 studies (14%) targeted athletes [22, 28, 33, 50, 65, 75, 83, 86, 91, 92, 93, 97]; 5 (6%) included runners [28, 83, 91, 92, 93], 3 (3%) included recreational athletes [33, 75, 86], 2 (2%) included unspecified athletes with a previous ankle sprain [50, 97], and 1 (1%) each for volleyball [65] and collegiate athletes [22]. Of the 87 studies, 7 (8%) included individuals with pes planus [43, 48, 51, 67, 71, 96, 102] and 6 (7%) post‐surgical patients [27, 57, 69, 90, 94, 98]. Four studies (5%) recruited college or university students [26, 47, 88, 99].

#### 3.1.2 Conditions Treated

Of the 87 studies, 31 targeted healthy individuals (36% of 87 studies) [4, 21, 28, 32, 37, 38, 43, 44, 48, 54, 58, 59, 64, 67, 68, 70, 71, 73, 77, 78, 83, 84, 85, 87, 91, 92, 93, 95, 96, 102]. Where specific conditions were targeted, ankle conditions were commonly chosen with 18 studies (20%) recruiting individuals with ankle instability [22, 26, 34, 35, 36, 45, 47, 52, 55, 56, 60, 65, 75, 76, 88, 97, 99, 100], 5 (6%) ankle fractures [27, 57, 69, 90, 98], and 4 (5%) acute and chronic ankle injuries [23, 24, 41, 50]. Tendinopathies were also targeted with seven studies (8%) including Achilles tendinopathy [31, 33, 42, 62, 63, 74, 103] and three (3%) tibialis posterior tendinopathy [40, 49, 81]. Eight studies (9%) provided exercise as an intervention for plantar heel pain [2, 25, 30, 39, 46, 79, 80, 101]. Less common conditions included hallux valgus in three studies (3%) [19, 53, 72], falls and balance issues in two (2%) [82, 94], and metatarsalgia [20], general foot pain [89], and posterior impingement [86] in one (1%) each. There were several studies that targeted more proximal conditions including patellofemoral joint pain in two studies (2%) [51, 66], lower limb [29], and low back pain [61] in one (1%) each.

### 3.2 Exercise Details

#### 3.2.1 Exercise Category

A total of 300 exercises were prescribed across all studies. The most common category of exercise was ankle plantar flexion exercises for 75 individual exercises (25%), followed by 50 exercises for plantar intrinsics (16%), 45 for ankle and digital dexterity (15%), and 27 for ankle dorsiflexion (9%). Ankle eversion (23, 8%) and ankle inversion exercises (22, 7%) had similar representation across all exercises prescribed. There were substantially fewer exercises targeted at the digits specifically: hallux (10, 3%) and lesser digits (1, < 1%). Other exercises included balance (15, 5%), plyometrics (3, 1%), and those classified in the other category (29, 10%) (Supporting Information S2).

#### 3.2.2 Primary Movement

Out of the 300 exercises, the most common primary movement featured in exercises prescribed across all studies was ankle plantar flexion (84, 28%), followed by digital flexion (39, 13%), ankle dorsiflexion (34, 11%), arch lifting (32, 11%), ankle eversion (23, 8%), ankle inversion (22, 7%), and digital abduction (4, 1%). There were 14 (5%) static exercises that did not elicit a movement (i.e., balancing exercises or voluntary isometric contractions) and 44 (15%) that used multiple movements in one exercise (i.e., tracing the alphabet with the foot and ankle or curling a towel with the digits). Less common movements were digital extension (2, < 1%) and digital adduction (2, < 1%) (Supporting Information S2).

#### 3.2.3 Exercise Prescription Variables

In relation to *frequency*, there was a large variation from once every hour (or hourly) to once per week. The most common frequency to perform exercises was three times per week for 101 of 300 exercises (34%), followed by daily (68 exercises, 23%), three times per day (20, 7%), five times per week (19, 6%), and two times per week (14, 5%). Other lesser prescribed frequencies included 30 min per week (9 of 300 exercises, 3%), every other day (3, 1%), once per week (4, 1%), 2 times per day (13, 4%), 2 to 3 times per day (8, 3%), 3 to 4 times per day (6, 2%), 4 times per day (2, < 1%), 4 to 5 times per day (4, 1%), hourly (2, < 1%), and a frequency was not reported 27 times (9%) (Supporting Information S2).

In relation to *intensity* of load, 198 of the 300 exercises (66%) did not have any specified type of load and 13 (4%) specified no load to be used. Of the remaining 89 exercises, 12 different types of load were prescribed. The most common equipment or objects to provide resistance or load were Thera‐Band, (51 of 300 exercises, 17%), followed by bodyweight only (11, 4%), elastic tubing (8, 3%), a weighted bag (5, 2%), dumbbells (4, 1%), pin‐loaded weights (2, < 1%), ankle weights (1, < 1%), the contralateral foot (1, < 1%), furniture (1, < 1%), gel toe separators (1, < 1%), and a Pilates reformer (1, < 1%) (Supporting Information S2).

A specified load intensity was not provided in 234 of the 300 exercises (78%). For the 66 exercises that it was provided, the load intensity, according to a participant's RM, was reported for 9 exercises (3%) [2, 39, 58, 78, 79, 80, 87]. Specifically, it was reported as a percentage of a 1RM for 1 exercise (< 1%) [58] or a percentage of 3RM for 2 exercises (< 1%) [87]. Alternatively, it was reported as a specified number RM without a percentage for 6 exercises (2%) [2, 39, 78, 79, 80]. Commonly, load intensity relative to an individual's RM was not reported, although a prescribed load was still provided for 56 exercises (18%). When a load intensity was prescribed not relative to a repetition maximum, the most common were red or medium Thera‐Band (23 exercises, 8%) and bodyweight (11 exercises, 3%). Other load intensities included varieties of Thera‐Band from extra heavy for seven exercises (2%) to light for two exercises (< 1%), a one to 2 kg (kg) weight for one exercise (< 1%), 3 kg weight for one exercise (< 1%), and no weight at all for six exercises (2%).

In relation to *progression*, 176 of the 300 exercises (59%) used progressions. Quantifiable progressions (i.e., using a prescribed quantity of load or volume) were used in 146 exercises. When quantifiable progression was used as part of an exercise prescription, the most common method was increasing load only (81 exercises out of 300, 27%), followed by volume only (28, 9%), volume then load (23, 8%), load then volume (12, 4%), volume or load (11, 4%), and volume and load (2, < 1%). Alternative progressions, such as changing the surface people, were using or giving people a choice of progression strategy, occurred in 8 exercises (3%), and an unspecified progression strategy occurred in 11 exercises (4%). It was unclear whether the alternative and unspecified progression offered by the program contributed to a load or volume increase or required a greater skill level to complete the exercise (Supporting Information S2).

In relation to *repetitions*, using a fixed number was the most common approach when prescribing exercises, which was used in 221 of the 300 exercises (74%). The most common repetition value used was 10 repetitions for 115 of 300 exercises (38%), followed by 15 repetitions (41, 14%), then 30 repetitions (19, 6%). Other repetition values included 5 (10, 3%), 8 (9, 3%), 12 (15, 5%), 20 (7, 2%), 100 (2, < 1%), and 200 (2, < 1%) repetitions. For one exercise, 26 repetitions [41] were used; this accounted for writing each letter of the alphabet once. Repetitions were not specified for 41 (13%) exercises (Supporting Information S2).

Some studies used a repetition range instead of a fixed number. Ten to 15 repetitions were the most common range (7, 2%), followed by 5–15 (4, 1%), 5–10 (2, < 1%), and 20–25 (1, < 1%) (Supporting Information S2). Other studies did not use a number or range, electing instead to use time or distance. The most common repetitions for time values were to perform an exercise for 10 min (8, 3%), followed by 3 min (2, < 1%), 1 min (2, < 1%), and 10 s (2, < 1%). All other timed approaches were only used once (< 1%), including 30 s, 45 s, 30–60 s, and 2, 4, 5, 6, 20, and 30 min (Supporting Information S2). One exercise prescribed a distance or length measure of a one‐m towel for toe scrunches (Supporting Information S2).

In relation to *sets*, most studies reported the number of sets for exercises. The most common number of sets prescribed per exercise was 3 sets across 110 of 300 exercises (37% of exercises), followed by a single set for 94 exercises (31%), 2 sets for 27 exercises (9%), 5 sets for 4 exercises (1%), 8 sets for 3 exercises (1%), and 4 sets for 3 exercises (1%). Ranged sets were also prescribed with ranges of two to three sets for seven exercises (2%), two to four sets for four exercises (1%), and one to three sets for two exercises (< 1%). Two studies prescribed one exercise each to be performed “as many [times] as possible” (< 1% of all exercises). Sets were not specified for 57 exercises, accounting for 19% of all prescribed exercises (Supporting Information S2).

### 3.3 Comparison of Exercise Prescription to the ACSM Guideline Recommendations

**FIGURE 2:**
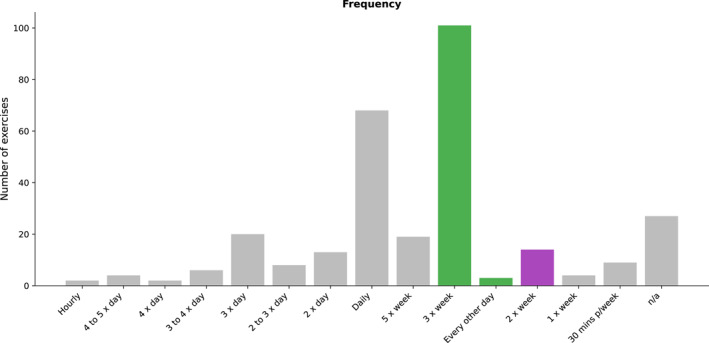
Frequency to perform individual prescribed exercises. Gray color indicates not meeting recommendations, green for meeting novice lifters, and purple for intermediate to advanced.

Firstly, we compared the training frequency of programs in the included studies to the ACSM guidelines [15] (Figure 2).

The recommended frequency for a novice or untrained individual in the ACSM guidelines is two to 3 days per week [15]. The frequency of exercise (i.e., how often to perform them) was reported 273 times across the 300 exercises (91% of all exercises). The prescribed frequency to perform an exercise ranged from once a week to hourly. The most common frequency was 3 times a week across 101 of 300 exercises (33%), which meets the novice and untrained lifter recommendations. Recreationally trained and athletic individuals are recommended to train less frequently at 2 days per week [15]. Training 2 times per week was prescribed for 14 of the 300 exercises (5%) and for once a week for 4 exercises (1%). Other frequencies prescribed per exercise of the 300 exercises included daily (69 of 300 exercises, 23%), not specified (27, 9%), 3 times a day (20, 6%), and 5 times a week (19, 6%). Aside from “not specified,” all of these prescriptions were more than any of the recommendations in the ACSM guidelines.

**FIGURE 3:**
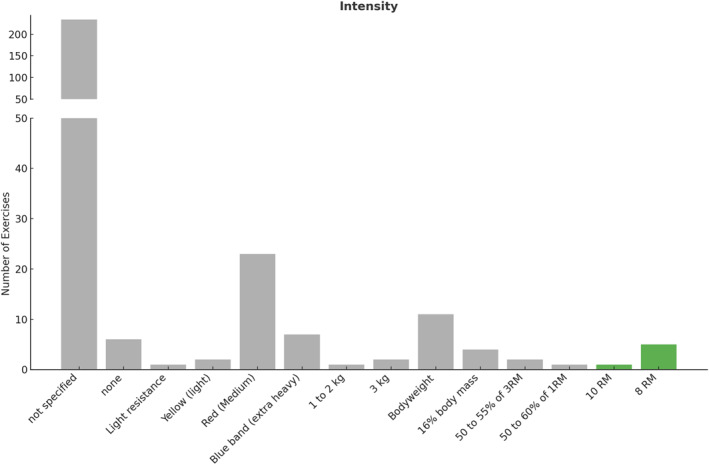
Load intensity of individual prescribed exercises. Gray color indicates not meeting recommendations, green for meeting novice lifters, and purple for intermediate to advanced.

Secondly, we compared the intensity of load prescribed in the studies included in this review to the recommendations provided by the ACSM guidelines [15] (Figure 3).

The guidelines report all load intensity according to a RM, as it provides a valid and consistent reference point for determining load intensity for an individual [104, 105]. The recommended load intensity for a novice or untrained lifter is 60%–70% of a 1RM (60%–70% 1RM) or 8 to 12RM [15]. None of the 87 studies included in this review reported a percentage of loading between 60% and 70% 1RM. However, an 8RM load intensity was reported for 5 of the 300 exercises (2%) [2, 78, 79, 80] and 1 study reported a 10RM for 1 exercise [39]. This load intensity would meet recommendations for novice lifters. One exercise was prescribed at 50%–60% of 1RM [58], and 2 were prescribed at 50%–55% of 3RM [87]; both are less than the recommendation for a novice lifter. The recommended load intensity for intermediate and advanced lifters is between 80% and 100% 1RM [15], which was not met in any study or for any exercise.

**FIGURE 4:**
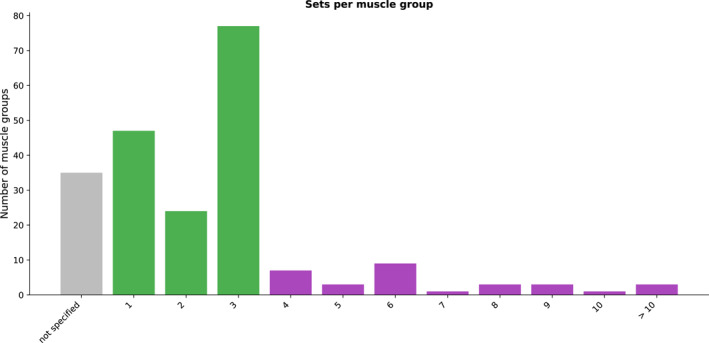
Sets per muscle group across all prescribed exercises. Gray color indicates not meeting recommendations, green for meeting novice lifters, and purple for intermediate to advanced.

Thirdly, the number of sets per muscle group prescribed in the studies included in this review was compared to the recommendations for strength training in the ACSM guidelines [15] (Figure 4).

To achieve optimal strength gains, the guidelines outline sets per muscle group and not per exercise. For this review, the muscle group was defined as a primary movement (e.g., plantar flexion and ankle inversion). According to the ACSM guidelines, the recommended number of sets per muscle group for novice lifters is between one and three [15]. In 28 of the 87 studies included in this review (32%), one set of exercises per muscle group was prescribed [4, 20, 27, 29, 30, 37, 41, 48, 51, 53, 57, 59, 60, 61, 64, 66, 67, 68, 73, 77, 89, 91, 92, 93, 94, 98, 100, 102]. In 16 studies (18%), 2 sets per muscle group were prescribed [4, 30, 35, 38, 41, 49, 53, 58, 66, 75, 77, 82, 92, 93, 97, 103]. The largest prescription per muscle group was 3 sets per muscle group, which was prescribed in 40 studies (46%) [2, 21, 22, 25, 32, 34, 35, 39, 41, 42, 43, 44, 45, 46, 47, 50, 52, 54, 56, 57, 62, 63, 65, 66, 70, 71, 75, 76, 78, 84, 85, 88, 89, 92, 93, 95, 96, 99, 101, 103]. For more experienced lifters, the ACSM guidelines recommend “*multiple sets to be used with systematic variation of volume and intensity over time*.” Of the 87 studies, 4 sets or more per muscle group were prescribed in 21 studies (30%) [4, 19, 20, 21, 28, 30, 31, 33, 34, 36, 40, 41, 49, 55, 66, 74, 86, 87, 89, 95, 97] with the number of sets ranging from 4 to 16. Of the 26 studies providing 4 or more sets, 4 of those studies specify athletes (i.e., advanced lifters) as participants [28, 33, 86, 97]. No specific sets were specified for 35 primary movements across 10 of the 87 studies (11%) [23, 24, 26, 69, 72, 79, 80, 81, 83, 90], with 2 of these studies prescribing “as many sets as possible,” which could achieve the recommendations for novice, intermediate, or advanced lifters [79, 80].

Finally, we assessed each program to assess how many met all recommendations for frequency, intensity, and time. For novice or untrained lifters, this would equate to 1 to 3 sets, at 60%–70% of a 1RM, 3 times per week. This was met in 5 of 87 studies (6%) [2, 39, 78, 79, 80]. For the intermediate or advanced lifter, this would equate to greater than 3 sets (as an increase in volume and intensity from a novice), at 80% or more of 1RM, 2 to 3 times a week. No programs met these recommendations.

### 3.4 Quality of Reporting

Of the 87 studies, the number of items reported on the CERT ranged from 1 to 15 (out of a maximum of 16). The median CERT score of all included studies was 5, equating to only 31% of the items on this checklist. Eighty‐three of the 87 studies (95%) provided a “detailed description of the exercise intervention including, but not limited to, number of exercise repetitions/sets/sessions, session duration, intervention/program duration, etc” [2, 4, 6, 19, 20, 21, 22, 24, 25, 26, 27, 28, 29, 30, 31, 32, 33, 34, 35, 36, 37, 38, 39, 40, 41, 42, 43, 44, 45, 46, 47, 48, 49, 50, 51, 52, 53, 54, 55, 56, 57, 58, 59, 60], 63 studies (72%) provided “detailed description of the type of exercise equipment (e.g., weights, exercise equipment such as machines, treadmill, and bicycle ergometer)” [2, 4, 7, 21, 23, 24, 26, 28, 29, 30, 31, 32, 33, 34, 35, 36, 37, 39, 42, 43, 44, 45, 46, 47, 48, 49, 50, 51, 52, 54, 58, 59, 61, 62, 63, 65, 66, 67, 68, 69, 70, 71, 72, 76], and 58 (66%) provided a “detailed description of the decision rule(s) for determining exercise progression and a detailed description of how the exercise program was progressed” [2, 4, 8, 21, 23, 25, 26, 33, 34, 35, 39, 40, 41, 42, 43, 44, 45, 46, 47, 48, 49, 51, 52, 54, 55, 56, 58, 59, 60, 61, 62, 63, 64, 65, 66, 68, 69, 70, 73, 75, 77, 78, 79, 80]. A “detailed description of any home program component (e.g., other exercises, and stretching)” was reported in 18 studies (24%) [23, 29, 33, 34, 37, 57, 61, 63, 66, 67, 69, 74, 79, 84, 87, 92, 93, 95] and a description of “whether there are any nonexercise components (e.g., education, cognitive–behavioral therapy, and massage,)” was reported in 8 studies (9%) [2, 4, 33, 37, 57, 79, 80, 87]. Seven studies (8%) provided sufficient information for the item “detailed description of motivation strategies” [2, 41, 42, 79, 80, 92, 93], “describe the type and number of adverse events that occurred during exercise” [4, 30, 42, 57, 58, 79, 80], and for “describe the decision rule for determining the starting level at which people commence an exercise program (such as beginner, intermediate, advanced, etc)” [2, 30, 70, 79, 80, 82, 85]. Sufficient information was provided for the item “Describe whether the exercises are generic (one size fits all) or tailored to the individual and detailed description of how exercises are tailored to the individual” in 20 studies (23%) [2, 4, 23, 30, 32, 45, 46, 49, 50, 58, 63, 66, 70, 78, 79, 80, 86, 89, 92, 93] and “how adherence or fidelity to the exercise intervention is assessed/measured and the extent to which the intervention was delivered as planned” in 17 studies (20%) [2, 21, 29, 30, 32, 34, 43, 45, 46, 47, 49, 50, 71, 74, 79, 80, 91]. No studies fulfilled all criteria (Supporting Information S3).

## 4 Discussion

Foot and ankle muscle strengthening exercises are a common intervention. They can be used for healthy populations to maintain adequate strength, although they are often prescribed by clinicians for managing lower limb musculoskeletal conditions [93, 106]. Our review set out to describe and critique the exercises outlined in the literature. Specifically, we aimed to (i) describe what foot and ankle strengthening exercises have been recommended in the scientific literature, (ii) compare these exercise prescriptions to the ACSM guidelines for prescription of strengthening exercises, and (iii) assess the completeness of the reporting of these exercises and exercise programs using the CERT.

### 4.1 Exercises Prescribed

For the studies included in our review, the ankle was the most commonly targeted region for strengthening exercises at the foot and ankle. Ankle plantar flexion exercises comprised 25% of the total exercises and were the most frequently prescribed exercise category and primary movement. This may be partly due to most conditions being related to ankle pathology. However, more than one third of the samples included healthy adults, demonstrating that most foot and ankle muscle strengthening programs, regardless of pathology, emphasize enhancing ankle plantar flexion movements. In addition, there was a high frequency of prescription of plantar intrinsic exercises, highlighting the significance attributed to intrinsic foot musculature. Interestingly, a relatively large percentage (15%) of dexterity exercises were prescribed, such as ankle alphabet (an exercise tracing letters using the ankle as the primary mover) and toe yoga (exercises using the digits to move independently of one another).

For *loading intensity* of strengthening exercises, studies used various types of resistance equipment, but red medium‐strength resistance bands (such as Thera‐Band) were found to be the most frequently used. Interestingly, while the use of resistance bands was a common method to provide resistance, no specified resistance or no resistance at all was used for most exercises. Achieving maximal strength gains requires adequate load intensity to stimulate change [10]. Inadequate load intensity has the capacity to not provide adequate stimulus to facilitate gains in strength [8, 107]. Prescribing inadequate loads is a consistent theme in this scoping review.

Exercise progression was incorporated into the majority of foot and ankle strengthening programs. This highlights that the investigators of the studies included in our review acknowledged that strengthening exercises require progression to achieve the best possible gains. This approach also aligns with the fundamentals of strength training and the recommendations outlined in the ACSM guidelines [15]. Most programs opted to increase load before increasing the volume, which is consistent with the ACSM recommendations that suggest increasing loads by 2%–10% for smaller muscle exercises (such as at the foot and ankle) when an individual can perform 2 or more repetitions beyond the initial prescription without reaching fatigue [15]. While load before volume may have been the most common, there was a large variety of approaches used to progress exercises across all studies, including increasing the perceived skill difficulty of an exercise and leaving the progression to a practitioner's discretion. Accordingly, further refinement and understanding of the effects of foot and ankle strength training exercise selection and prescription protocols on foot and ankle musculature should be a priority for future research.

For *time*, a diversity of approaches was used, but fixed repetition was by far the most common prescription method. Sets and repetitions were often prescribed as 3 sets of 10 repetitions per exercise. However, this prescription is a 'one size fits all’ or generic approach and may not consider strength training fundamentals such as specificity or variation for optimum benefits at the foot and ankle [105]. Furthermore, like all strength training protocols, this prescription structure was designed for training the entire body (including large muscle groups such as the hips or chest) and has been adapted for use with proximal structures rather than being specifically designed for achieving maximal strength gains at the foot and ankle.

### 4.2 Comparison to Best Practice

The ACSM guidelines have established dosage recommendations for strengthening exercise prescriptions across different populations [15]. For novice lifters (with some mild reduction in volume for certain older populations), it is recommended that optimal strength gains occur when training 4 sets per muscle group (or primary movement), using 60%–70% 1RM with 2–3 training sessions per week [8, 9, 10, 15]. For intermediate and advanced lifters, higher load intensity and an increase in training volume are recommended for increased strength gains with the ACSM recommending advanced individuals cycle loads of 80% 1RM or greater [15]. These recommendations are supported by several meta‐analyses to estimate the optimal number of sets and load intensity for beginners, the recreationally trained, and athletes to achieve strength gains [8, 9, 10].

The prescription of training frequency, in the studies included in our review, often exceeds best practice recommendations, with some exercises prescribed excessively (e.g., hourly and daily) compared to the ACSM guidelines of three times per week for a novice and less than that for advanced lifters [15]. While frequency may impact strength gains less than load intensity or set volume, adequate recovery time is an important programming consideration for strength adaption [108]. Increasing frequency must always be viewed in the context of the collective volume of load intensity, sets, and exercise selection [15]. Although occasionally, high frequency training might be beneficial, several meta‐analyses have shown that the highest yield of strength gains occurs at two times per week for advanced individuals [8, 10].

Regarding load intensity, higher loads are needed to achieve optimal strength gains compared to hypertrophy or muscular endurance [8, 11]. However, load intensity, in relation to a RM, was only reported in 3% of exercises in our review. Repetition maximum is not only a valid measure to determine an individual's strength capacity, but also the most appropriate method to determine the prescription of load intensity for that individual [109, 110, 111]. There may be some balance exercises (e.g., single leg balance) where it is difficult to determine a load based on RM, but balance exercises only accounted for 5% of the exercises included in our review. When loads were reported, they rarely met the ACSM guidelines, with 2% of exercises reporting a load intensity that would be considered acceptable for novices or for more frail populations aged over 65 years (i.e., between 60% and 80% 1RM) and none meeting recommendations for intermediate or advanced lifters (i.e., 80% RM or greater) [15]. Moreover, load intensity was infrequently reported. Load was not reported in 76% of exercises; although we cannot be certain, some possibly used bodyweight only. If this was the case, these exercises may have been under‐loaded (or over‐loaded) to optimize for strength gains compared to the recommendations outlined by the ACSM guidelines. Furthermore, many exercises provided resistance bands and weights under 3 kg, which could also represent loads that do not meet recommendations. As the ankle joint complex can produce a force up to five times an individual's body weight during running, this amount of load is likely to be inadequate [112]. Future studies could avoid this by prescribing loads as a percentage of RM at an appropriate load intensity, as suboptimal loads will not lead to maximal strength gains.

Most programs met the set volume recommendations for novice lifters, with 62% prescribing between 1 and 3 sets per muscle group. Considerably, fewer studies included programs with four or more sets. The investigators concluded that athletes (advanced lifters) should consider increases in volume up to eight sets per muscle group to achieve optimal strength gains [8, 9, 10]. This is a significant increase in volume when compared with the recommendations for novice lifters [8, 9, 10], but is consistent with the recommendations set by the ACSM guidelines [15]. However, of the 12 studies that recruited athletes included in our review, only 2 prescribed 8 or more sets for a primary movement [28, 86]. Therefore, in the majority of studies, the advanced lifter or athletic population was prescribed inadequate volume to optimize strength gains. Regarding the 12 studies designed for the older populations, the prescriptions were between 1 and 6 sets per muscle group, which is consistent with all the other studies aimed at healthy adults that ranged from insufficient to more than recommended for older or more frail populations [15]. There is an opportunity that researchers consider prescribing a greater volume of sets per primary movement to optimize outcomes for strength gains, especially if targeting athletic populations. This may also be the case for clinicians if they are basing their prescriptions on current scientific literature, that is, the studies included in our review.

Finally, only 5% of the studies met all ACSM recommendations for a training program for novice lifters in relation to sets, load intensity, and frequency. These programs prescribed 3 sets per primary movement (plantar flexion), with loads of 8 to 10RM, performed 3 times per week [1, 2, 3, 4, 5]. By meeting the needs of novice lifters, these recommendations may better represent the requirements of the general population. However, the small percentage of studies meeting these guidelines highlights that most foot and ankle strengthening programs in the studies included in our review did not meet recommendations for optimal strength gains.

Overall, while all strength training yields positive outcomes, adherence to best practice principles can optimize strength gains [10, 11]. Discrepancies between what was prescribed in the studies included in our review and best practice suggest that more tailored and informed strengthening programs should be used for the foot and ankle to optimize strength gains, with a focus on increasing load intensity (i.e., % RM) as the priority.

### 4.3 Quality of Reporting

As previously found [12, 13, 14], exercises were generally poorly reported. We found that nearly all studies included in our review reported a name for each exercise. In more than half of the studies, the names of exercises were synonymous with how the exercises are performed. However, many of the exercises were difficult to understand due to the complexity of the description. For example, the “short foot exercise” is challenging to explain and demonstrate. Indeed, due to its complexity, this exercise also is also referred to by other names, including “doming” and “plantar arch raise,” and the descriptions of how to perform the exercise also varies widely. Certainly, many exercise names do not create a clear understanding of how to perform the exercise. This raises the question of whether simply providing a name or description of the exercise is sufficient. Supporting images and videos of how an exercise is performed would be helpful, which has previously been suggested by Christensen et al. [12]. A package of information that includes consistent naming and descriptions of specific exercises that also has accompanying audio and/or visual information would likely provide optimal information for an individual to successfully perform the exercises. While this would be worthwhile for patients, it would also be helpful to improve consistency and repeatability in research.

The establishment of a tailored approach to an exercise program was generally under reported. When considering a tailored approach, future studies may consider tailoring prescription variables to individuals' capacity using a % of RM rather than giving everyone the same prescribed load (i.e., 3 kg or medium Thera‐Band). Such an approach would allow for each program to be adequately tailored for each participant, according to their capacity or pain levels. It would also be easier for clinicians to translate the program into practice and would provide some basis to determine if the program requires modification based on a patient's level of discomfort. This could easily be achieved by determining a participant's RM for any given weight at the start of the program and provide an adapted weight relative to the participant's %RM prescribed. Further, this RM method can be translated to different equipment, from Thera‐Bands to dumbbells, and is not a new concept to strength and conditioning literature. The authors acknowledge that the RM method has only been established for bench press, squat, and deadlift, but it has not yet been established for foot and ankle exercises [111]. Nonetheless, the principle can still be applied to the foot and ankle until more research is conducted. Based on this, the resistance (or load) can be tailored for an individual's capacity (i.e., use more or less resistance or load), and doing so, increases the likelihood that the prescription, including the progression of load, is optimized to achieve strength gains.

When considering conditions of the foot and ankle such as plantar heel pain and tendinopathies, everyday walking and other loads contribute to the cumulative load on tissues [113]. Therefore, the inclusion of the reporting item “whether there are any home program” or “nonexercise components” is useful in understanding the overall loads to which participants are subjected. These items are important as additional loads, outside of those applied in a strength training program, may negatively influence pain and function [114]. Accordingly, we recommend that future studies include a statement about how much incidental exercise or other loading exercises may or may not be included to provide a more complete indication of the entire loading regime.

With the above in mind, our findings indicate that future research offering strength training as an intervention should include detailed descriptions (with online video instructions likely being even more effective), including dosage of exercises and programs for completeness, transparency, and optimal benefit.

### 4.4 Strengths and Limitations

There are several strengths of our review, which collated 87 studies and 300 exercises to summarize the literature of foot and ankle strengthening exercises. The review includes studies from various countries and covers a range of conditions, providing a broad perspective on what is currently being prescribed. It also compared their exercise prescriptions to best practice guidelines and evaluated the reporting of these exercises. We believe this is the first review of this kind to be published.

However, our review has a number of limitations that need to be considered. The authors acknowledge that only two databases have been searched, and relevant studies may have been missed. However, to account for this, hand searching of reference lists, gray literature, and studies included in systematic reviews was performed.

Only one author performed all screening and data extraction of included articles and data. Additional reviewers would have improved the rigor of the search, screening and data extraction process. Future reviews should consider two or more reviewers to screen and extract data.

Exercise prescription is complex and can include many variables that were not assessed in our review (e.g., rest, time between sets, and exercise tempo). Instead, we sought to focus on the key variables of sets, repetitions, and frequency as they are common prescription variables used by researchers and clinicians.

The ACSM best practice guidelines were developed for the whole body and were not specific to the foot and ankle. Understanding the impact of exercise choice and its prescription variables (sets, loading magnitude, and training frequency) on the foot and ankle specifically would be beneficial.

A recommendation of our review is to use the CERT as a method for reporting exercise, which aligns with previous research [13, 14]. However, we are aware that one study [12] has suggested that the CERT lacks enough specific details to allow adequate translation to clinical practice.

Finally, the ACSM guidelines and CERT checklist were published after many of the studies in our review were conducted, so the low adherence to these was not unexpected.

## 5 Conclusion

This review found that prescriptions of foot and ankle muscle strengthening exercises predominantly recommend exercises targeted at the ankle plantar flexion and intrinsic muscles, typically prescribed at 3 sets of 10 repetitions 3 times a week, often using no prescribed load. In addition, foot and ankle exercise programs do not provide adequate load intensity to maximize strength gains when compared to recommendations from the ACSM. There are also substantial deficiencies in the reporting of foot and ankle exercises, making them difficult to understand and implement. We suggest adopting established best practice recommendations like the ACSM guidelines for exercise testing and prescription and the CERT for improved exercise reporting.

## Author Contributions

**John W. A. Osborne:** conceptualization, formal analysis, investigation, methodology, visualization, writing–review and editing. **Hylton B. Menz:** conceptualization, formal analysis, investigation, methodology, writing–review and editing. **Glen A. Whittaker:** investigation, methodology, writing–review and editing. **Matthew Cotchett:** investigation, methodology, writing–review and editing. **Karl B. Landorf:** conceptualization, formal analysis, investigation, visualization, writing–review and editing.

## Ethics Statement

The authors have nothing to report.

## Consent

The authors have nothing to report.

## Conflicts of Interest

H.B.M. is the Emeritus Editor and Professor K.B.L. is a member of the Editorial Board of the Journal of Foot and Ankle Research. It is a journal policy that editors are removed from the peer review and editorial decision‐making processes for manuscripts they have coauthored.

## Supporting information

Supporting Information S1

Supporting Information S2

Supporting Information S3

## Data Availability

The data analyzed during this study are available from the corresponding author upon reasonable request.
