# Supplementary material for: Muscle Strengthening Exercises for the Foot and Ankle: A Scoping Review Exploring Adherence to Best Practice for Optimizing Musculoskeletal Health
Source: J Foot Ankle Res. 2025 Apr 3;18(2):e70040. doi: 10.1002/jfa2.70040 (PMC11967365; doi:10.1002/jfa2.70040)
Supplement: Supplementary file 1 — Supporting Information S1 [file JFA2-18-e70040-s001.docx]

**Supplementary file 1** Systematic search strategy for Medline and CINAHL

| Medline | | CINAHL | |
| --- | --- | --- | --- |
| 1. | Exercise/ | 1. | ‘muscle strengthening exercise’ OR ‘rehabilitation exercise’ OR ‘short foot exercise’ |
| 2. | Resistance training/ | 2. | foot OR ankle OR foot arch OR toe |
| 3. | ‘Strength training’ | 3. | 1 AND 2 |
| 4. | ‘Weight-lifting exercise program’ |  |  |
| 5. | ‘Weight-lifting strengthening program’ |  |  |
| 6. | Foot/ |  |  |
| 7. | Ankle/ |  |  |
| 8. | ‘Toe’ |  |  |
| 9. | 1 OR 2 OR 3 OR 4 OR 5 |  |  |
| 10. | 6 OR 7 OR 8 |  |  |
| 11. | 9 AND 10 |  |  |

The search strategy used for systemic search of the literature. All terms are outlined in order of how search was conducted.
